# Chromatographic Studies of Protein-Based Chiral Separations

**DOI:** 10.3390/separations3030027

**Published:** 2016-09-05

**Authors:** Cong Bi, Xiwei Zheng, Shiden Azaria, Sandya Beeram, Zhao Li, David S. Hage

**Affiliations:** Department of Chemistry, University of Nebraska-Lincoln, Lincoln, NE 68588-0304, USA

**Keywords:** chiral high-performance liquid chromatography (HPLC), protein-based chiral stationary phases, chiral recognition, chromatographic studies of drug-protein interactions, frontal analysis, zonal elution

## Abstract

The development of separation methods for the analysis and resolution of chiral drugs and solutes has been an area of ongoing interest in pharmaceutical research. The use of proteins as chiral binding agents in high-performance liquid chromatography (HPLC) has been an approach that has received particular attention in such work. This report provides an overview of proteins that have been used as binding agents to create chiral stationary phases (CSPs) and in the use of chromatographic methods to study these materials and protein-based chiral separations. The supports and methods that have been employed to prepare protein-based CSPs will also be discussed and compared. Specific types of CSPs that are considered include those that employ serum transport proteins (e.g., human serum albumin, bovine serum albumin, and alpha_1_-acid glycoprotein), enzymes (e.g., penicillin G acylase, cellobiohydrolases, and α-chymotrypsin) or other types of proteins (e.g., ovomucoid, antibodies, and avidin or streptavidin). The properties and applications for each type of protein and CSP will also be discussed in terms of their use in chromatography and chiral separations.

## 1. Introduction

The use of molecular chirality can be traced back to the mid-1800s in the work by Louis Pasteur with tartaric acid [1–3]. In the late 1970s, Yoshio Okamoto synthesized a helical polymer of triphenylmethyl methacrylate and used this material to develop chromatographic columns that could be used for chiral separations [4]. Since that time, significant effort has been aimed at creating chiral stationary phases (CSPs) that can be used in chromatography for the separation and analysis of chiral compounds [3–9].

Many pharmaceutical agents are chiral molecules [7,10–13]. Although these compounds are often sold as racemic mixtures, about two-thirds of the drugs that are currently available commercially are chiral [7,14]. Even though the two enantiomers of a drug will have most of the same physical properties, they may vary significantly in how they interact with a biological system, possibly leading to different levels of activity or side effects [10,11,15]. For example, *S*-ibuprofen is over 100 times more potent than *R*-ibuprofen at cyclooxygenase 1 and 2 inhibition, and *R*-thalidomide is a sedative while *S*-thalidomide is a teratogen. In addition, the *S*-enantiomers of barbiturates such as pentobarbital, hexobarbital, and mephobarbital are sedatives/hypnotics while the *R*-enantiomers tend to be inactive [10,15]. These differences have made it necessary to provide pure enantiomers in pharmaceutical formulations and/or to determine the effects the separate enantiomers may have in the body [7,9]. This, in turn, has led to the need for analytical methods and preparative techniques that can be used to analyze and separate chiral compounds in pharmaceutical samples [7,9].

Several methods have been employed for the chiral separation of drugs and amino acids for chemical analysis. These methods have included gas chromatography, thin layer chromatography, high-performance liquid chromatography (HPLC), supercritical fluid chromatography, and capillary electrophoresis [6,9,16–18]. Methods based on HPLC have been of particular interest [6,9,17–19]. Many types of stereoselective binding agents have been used to prepare CSPs for HPLC. The use of these agents in a liquid chromatographic system can be viewed as a type of affinity chromatography or high-performance affinity chromatography (HPAC) [3–9]. Examples of such binding agents have included proteins, polysaccharides, macrocyclic antibiotics, and ion-exchange ligands, among others [5,6,17,19–21].

Many proteins have the potential to discriminate between the separate forms of a chiral compound. This is the case because proteins are also chiral in nature in terms of both their primary structure (i.e., being made of L-amino acids) and their secondary or higher order structures [17]. Various types of proteins have been immobilized and used to make CSPs for HPLC, ranging from enzymes to serum transport proteins and glycoproteins. Some common examples of these proteins are shown in Table 1 [17,18,20–26]. These separations are performed based on differences in the binding strength and amount or number of binding sites for chiral compounds with the protein [3]. Advantages of using protein-based CSPs include their tendency to be able to work with a broad range of chiral compounds, their ability to be used with aqueous mobile phases, and their ability to directly analyze most chiral compounds without the need for derivation [17,19,22,27]. However, protein-based CSPs can also have a low loading capacity, be expensive to prepare, and have limited stability, which makes these of greater interest for analytical work than for preparative separations [17,19,28,29].

This review will discuss how chromatographic methods have been used to examine and characterize protein-based chiral separations. Tools that have been used in such work will first be considered, including zonal elution, frontal analysis, and kinetic methods of analysis. Methods and supports that have been employed for preparing protein-based CSPs will then be considered. The properties and applications for several specific types of protein-based CSPs will then be examined. This will include CSPs that employ various types of serum transport proteins and enzymes, as well as binding agents such as antibodies and avidin/streptavidin. The relative advantages for each type of CSP will be discussed, along with information that has been obtained through chromatography on these materials and their ability to bind and separate chiral agents.

## 2. Chromatographic Methods Used to Study Protein-Based Chiral Separations

### 2.1. Zonal Elution

**Figure 1:**
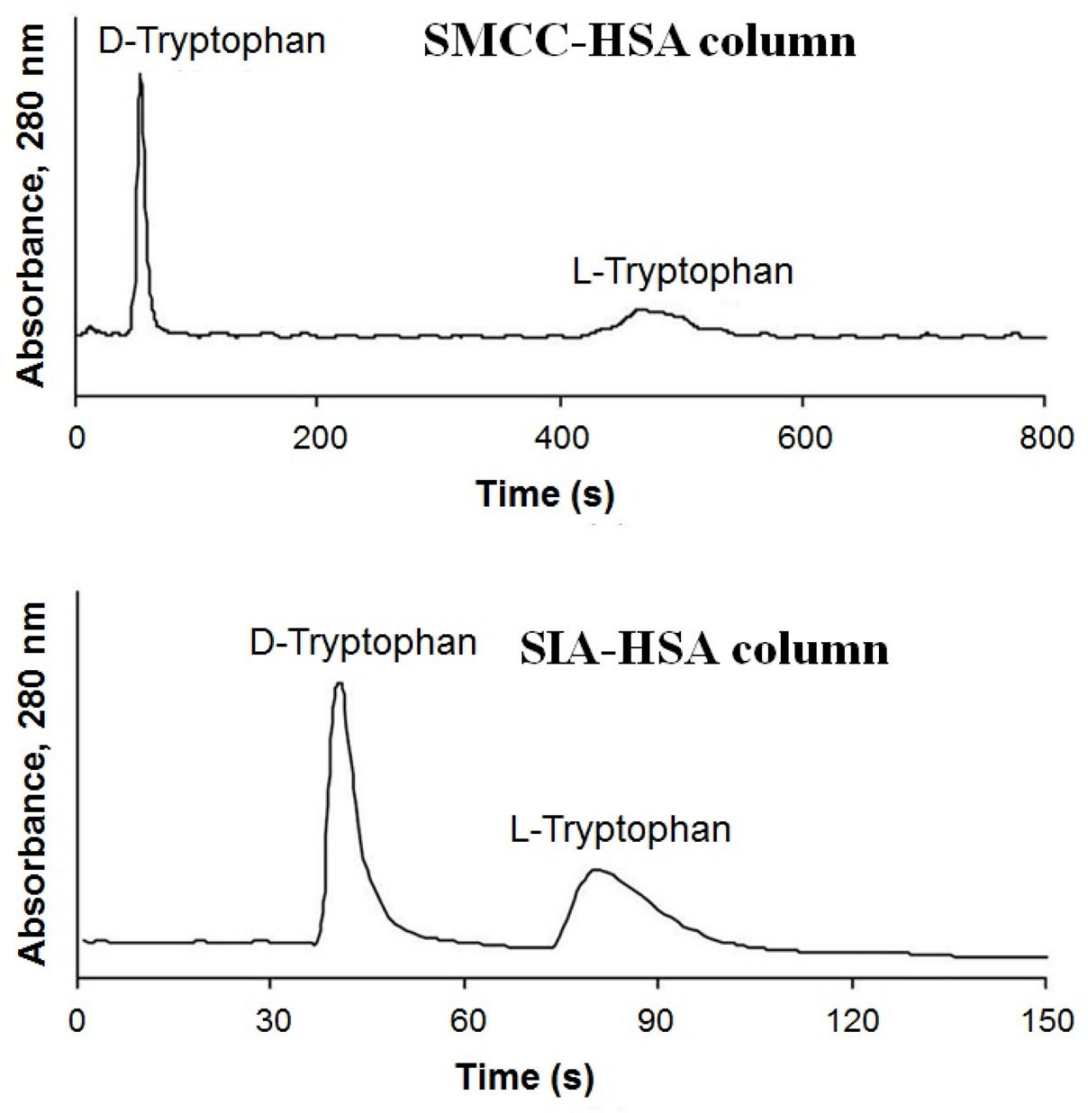
Chromatograms obtained by zonal elution for the chiral separation of D- and L-tryptophan on high-performance liquid chromatography (HPLC) columns containing human serum albumin (HSA) immobilized to sulfhydryl-reactive silica that had been activated by using succinimidyl 4-(*N*-maleimidomethyl) cyclohexane-carboxylate (SMCC) or succinimidyl iodoacetate (SIC). Reproduced with permission from [35]. Copyright American Chemical Society, 2007.

Zonal elution has been widely used in HPLC-based chiral separations [30–32]. This method can also be employed to measure the separation factors and resolution of solutes on CSPs (see Figure 1) [33–35]. Another use of zonal elution is to obtain information on the interactions between an injected solute and an immobilized protein. This approach can provide information on the strength of solute–protein binding, the changes in binding that occur as the mobile phase composition or temperature is changed, the location and types of binding sites for the solute on the protein, and the effect of altering the solute or protein structures on the interactions of these agents [32,36,37]. Zonal elution can be used to provide this information when a small amount of the solute of interest is injected onto a protein-based CSP in the presence of a competing agent or a mobile phase with a known composition and temperature. The retention factor for the solute is then measured and the change in this value is examined as the conditions used within the column are varied [38,39].

For a solute or analyte (A) that is injected onto a column that contains an immobilized protein, the retention factor for the analyte (*k*) can be related to the number of binding sites for the analyte on the protein and the equilibrium constants for the analyte at these sites. Equation (1) is an example of this relationship for a protein-based CSP with a series of independent binding sites (1 through *n*) for the analyte [40].

(1) $$k=(K_{A1}n_{1}+\cdots +K_{An}n_{n})m_{L}/V_{M}$$

In this equation, the terms *K*_A1_ through *K*_A_*_n_* are the association equilibrium constants for the analyte at each type of binding site in the column, *n*_1_ through *n_n_* represent the relative amount or moles for each type of site, *m_L_* is the total moles of binding sites that are present, and *V_M_* is the column void volume. According to Equation (1), the retention of the analyte can provide both qualitative and quantitative information on a solute–protein interaction. This includes the relative amount of active binding sites in the column (i.e., *m_L_*/*V_M_*) and the overall binding strength of these sites (i.e., *K*_A1_*n*_1_ + … + *K*_A_*_n_n_n_*) [32]. The advantages of using zonal elution to obtain this data include its need for only a small amount of the analyte and its ability in some cases to study more than one analyte per injection [39]. In addition, this method can be used with standard HPLC systems and often requires only short analysis times [39].

Another application of zonal elution has involved the development of quantitative structure-retention relationships (QSRRs) that characterize the binding of drugs and other solutes to protein-based CSPs [41–43]. In this type of study, a set of retention parameters are acquired for a series of analytes and compared to various structural descriptors for these solutes to see which of these factors influence retention [42]. The resulting QSRRs can then be used to predict the retention of a related solute, to investigate the molecular mechanism of the chiral separation, or to compare the retention properties of CSPs [42]. Specific applications of this approach have involved its use to examine the retention of benzodiazepine derivatives on human serum albumin (HSA) columns [43], to study the stereoselective separations of chiral solutes on a bovine serum albumin (BSA) column, and to characterize the chiral recognition of columns produced with BSA fragments [41].

Competitive binding studies can be used in zonal elution to determine which types of sites are binding to a given form of a drug and to estimate the equilibrium constants at these individual sites [34,44–47]. In this approach, a small amount of the analyte is injected onto a column that contains a protein (or other type of binding agent) and in the presence of mobile phases that have various known concentrations of a competing agent, I [34,44,45]. The retention factor observed for the analyte (*k*_A_) should change as the concentration of the competing agent, [I], is varied if both A and I are binding to the same sites in the column. For instance, Equation (2) shows how *k*_A_ will change with [I] for a system in which A and I have competition at a single type of site and A has no other sites in the column [34,44,45]:

(2) $$\frac{1}{k_{A}}=\frac{K_{I}V_{M}[I]}{K_{A}m_{\mathrm{Ltot}}}+\frac{V_{M}}{K_{A}m_{\mathrm{Ltot}}}$$

In this equation, *K*_I_ or *K*_A_ is the association equilibrium constant for the binding of I or A at their common site, and *m_Ltot_* is the total moles of active binding sites at which A and I compete [34,44,45]. This method has been used to examine the interactions between D- and L-tryptophan on HSA, as well as the interactions between these amino acid enantiomers and *R*-warfarin [45]. Related equations have been employed to examine multisite binding and allosteric interactions between drug enantiomers and other drugs or solutes on HSA columns [44,48–52].

Another use of zonal elution has been to evaluate the efficiency of protein-based CSPs, as can be done through band-broadening measurements [53–58]. It has been found that the efficiency of these columns can be affected by factors such as temperature, the amount of immobilized protein, the flow rate, and the mobile phase composition [53–57]. This approach has been used with HSA monoliths to study and optimize the chiral separation of *R*- and *S*-warfarin or D- and L-tryptophan [54,56]. This approach has also been utilized to examine the chiral separation for *R*- and *S*-warfarin or *R*- and *S*-propranolol on various columns containing alpha_1_-acid glycoprotein (AGP) [54,56].

### 2.2. Frontal Analysis

**Figure 2:**
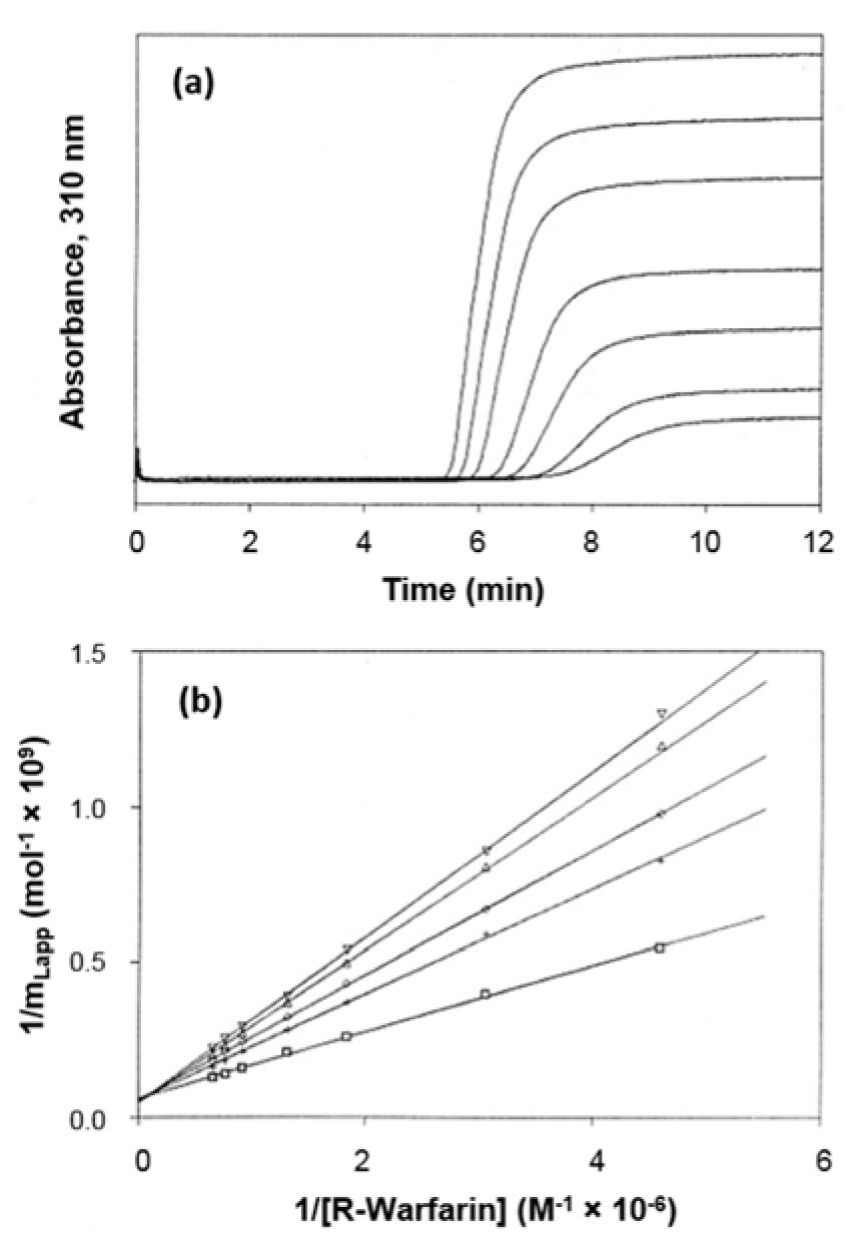
Frontal analysis results obtained for *R*-warfarin on an immobilized human serum albumin (HSA). The breakthrough curves in (**a**) were obtained at 4 °C and using *R*-warfarin concentrations (from left-to-right) of 1.50, 1.30, 1.10, 0.76, 0.55, 0.33 and 0.22 μM. The plots in (**b**) were obtained when such data were acquired at various temperatures (4–45 °C) and analyzed according to a double-reciprocal form of Equation (3). Reproduced with permission from [65]. Copyright American Chemical Society, 1994.

Frontal analysis is another chromatographic approach that has been used to examine the chiral interactions of drugs and other solutes with proteins [31,33,59]. In this method, a solution containing a known concentration of an analyte is continuously applied to a column that contains an immobilized protein or binding agent. The amount of the analyte that passes through the column is then monitored as a function of the amount of time or mobile phase volume that is used for application of the analyte [32,60,61]. As binding sites in the column become occupied by the analyte, a breakthrough curve is observed (see Figure 2) [32,60,61]. This experiment is repeated at several known concentrations of the analyte. If fast association and dissociation are present between the analyte and the protein, the mean position of each breakthrough curve (i.e., the breakthrough time or volume) can be used to obtain information on the thermodynamics of the interaction. This is done by fitting the results to binding models, such as those given in Equations (3) and (4) [32,62,63]:

(3) $$m_{\mathrm{Lapp}}=\frac{m_{\mathrm{Ltot}}K_{a}[A]}{1+K_{a}[A]}$$

(4) $$m_{\mathrm{Lapp}}=\frac{m_{L1}K_{a1}[A]}{1+K_{a1}[A]}+\frac{m_{L2}K_{a2}[A]}{1+K_{a2}[A]}$$

Equation (3) describes the interaction of analyte A with a single type of saturable binding site, in which *m_Ltot_* is the total moles of binding sites that are present for the analyte, and *K_a_* is the association equilibrium constant for the analyte at these sites [32,60,61]. Equation (4) applies to a case in which the column contains two groups of saturable sites for the analyte. In this latter situation, *m_L_*_1_ and *m_L_*_2_ are the total moles of sites 1 and 2, respectively, and *K_a_*_1_ and *K_a_*_2_ are the association equilibrium constants for the analyte at these sites [32,60,61]. Such models have been applied in the investigation of the equilibrium constants and number of binding sites for many chiral drugs with proteins such as HSA and AGP [34,45,57,64,65]. The same type of approach can be utilized with other binding models, as has been demonstrated in frontal analysis studies that have examined the interactions of *R*- and *S*-propranolol with low density lipoprotein and related binding agents [66–69].

An advantage of using frontal analysis to study protein-based CSPs is that this method can simultaneously provide information on both the equilibrium constants and the number of binding sites that are involved in the interaction of a solute with this type of column [32,60,61]. Frontal analysis can also be used with various types of supports and detection methods, including those used in either low- or high-performance liquid chromatography [32]. However, this approach does tend to require a larger amount of solute than zonal elution and often takes longer to perform because of the need for experiments at multiple concentrations of the analyte [32].

To characterize a protein-based CSP, frontal analysis is typically used to study the binding by separate chiral solutes to the column. The information that is obtained for the various chiral forms can be used to examine the retention mechanism and optimize the separation of these forms on the CSP [6,31,57]. Examples of such work include frontal analysis studies that have been conducted with *R*- and *S*-warfarin or D- and L-tryptophan on HSA columns (see Figure 2) [57,65]. Frontal analysis was utilized in these two cases to determine the binding strength and amount of binding sites for each enantiomer on the protein-based CSPs. Based on this information, it was possible to predict the behavior of these analytes during chiral separations [57,65].

### 2.3. Kinetic Studies

The rate of a solute–protein interaction is another factor to consider in characterizing the separation of chiral drugs. This information can be helpful in examining the mechanism of a protein-based chiral separation and in optimizing such a separation [53,70–72]. Several chromatographic approaches have been used to examine the kinetics of drug–protein interactions. These methods include plate height measurements and the peak decay method [53,70–73].

**Figure 3:**
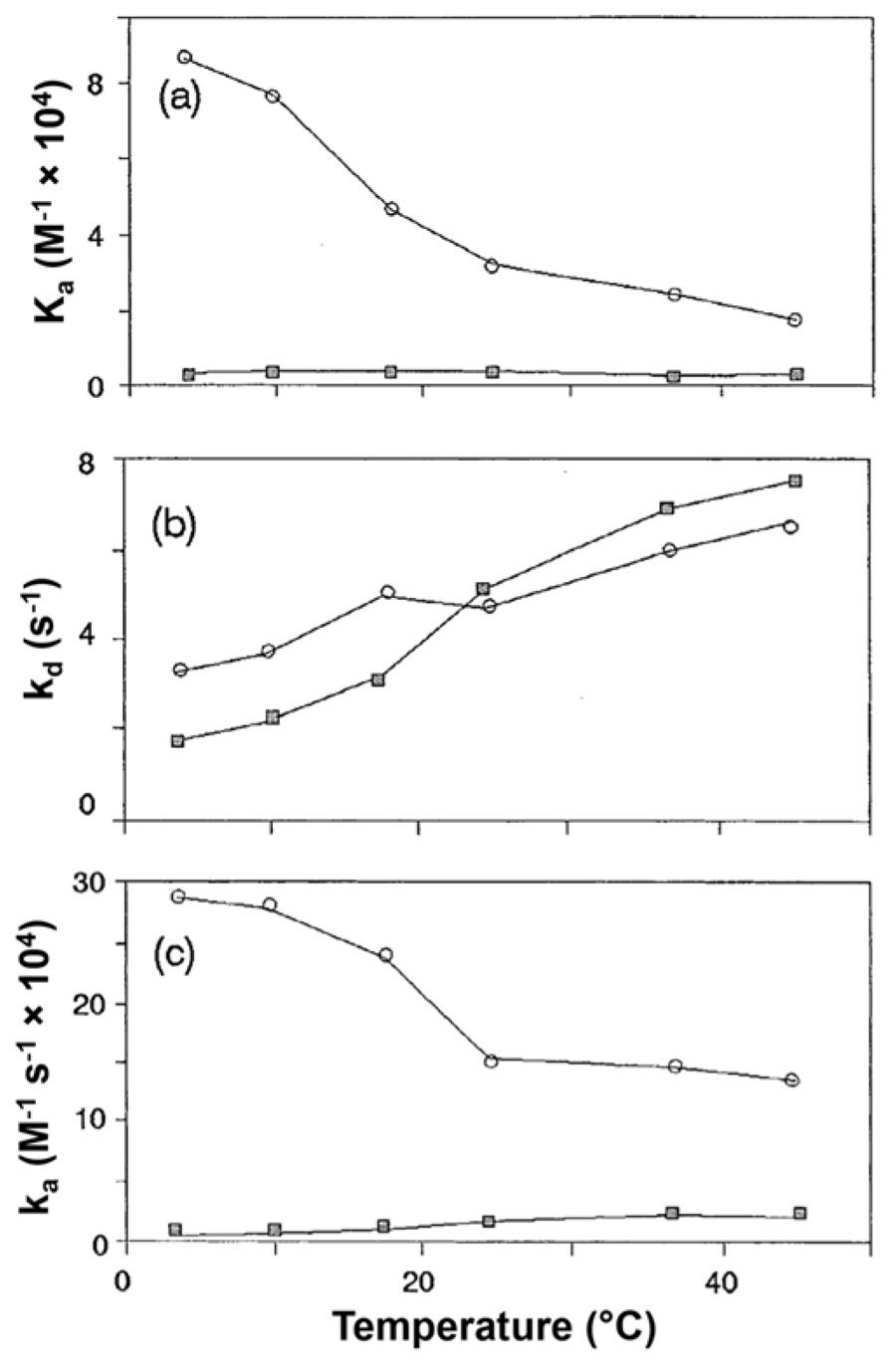
Use of frontal analysis and band-broadening measurements to measure the effect of temperature on the (a) association equilibrium constants (*K_a_*), (b) dissociation rate constants (*k_d_*) and (c) association rate constants (*k_a_*) for the interactions of D-tryptophan (■) and L-tryptophan (○) with a column containing immobilized human serum albumin (HSA). Reproduced with permission from [53]. Copyright Elsevier, 1997.

The plate height and band-broadening measurements have been used in several studies to examine the rate of a drug interaction with an immobilized binding agent such as a protein [53,71–76]. For instance, plate height measurements have been used to obtain the dissociation and association rate constants for *R-* and *S*-warfarin or D- and L-tryptophan on columns containing immobilized HSA (see Figure 3) [53,72]. This kinetic information was obtained as conditions such as the mobile phase composition and temperature were varied and were used to optimize chiral separations for the given solutes on HSA columns. The temperature data were further employed to determine the changes in enthalpy, entropy, and total free energy that occur during the formation of an activated complex between each enantiomer and HSA [53,72]. Plate height measurements have also been utilized in a closely related method known as peak profiling to examine the interactions and separation of chiral phenytoin metabolites on an HSA column [71].

The peak decay method is another technique that has been used to study the kinetics for chiral drugs on columns containing immobilized proteins [73,76–78]. In this method, the target compound is first applied to the column, followed by a switch to flow rate and mobile phase conditions that allow elution of this target as soon as it dissociates from the immobilized binding agent. If this experiment is carried out under the proper column size and flow rate conditions, release of the bound target from the column should follow a pseudo-first-order decay that can be used to obtain the dissociation rate constant for the target from the immobilized binding agent [73,76–78]. A noncompetitive peak decay method was used with small HSA columns to examine the dissociation rate of *R*- and *S*-warfarin [70]. This work included an evaluation of how factors such as the column size, the amount of applied analyte, the flow rate, and particle size of the support affected these measurements [70]. In addition to drug enantiomers, the peak decay method has been used to determine the dissociation rates for drugs such as imipramine, cisplatin, and lidocaine from HSA or AGP [70,78,79].

## 3. Preparation of Protein-Based CSPs

### 3.1. Supports Used in Protein-Based CSPs

Various support materials have been employed in protein-based CSPs. Examples of these materials are agarose, silica, polymeric supports, and zirconia particles [18]. Agarose was used as early as 1973 to immobilize BSA for chiral separations [17,80]. Silica particles and related materials have also been used as supports in protein-based CSPs. The efficiency and good mechanical stability of these supports have been valuable in creating CSPs for use in HPLC systems [3,17,18]. Silica is generally limited to a pH range of 2–8 in these applications; however, this has not been a major issue in work with protein-based CSPs, which are often used at a neutral pH or over a pH range of 3–8 [17]. Silica monoliths have also been considered for use in protein-based chiral separations [54,59,81,82]. This type of monolith can be obtained commercially and is prepared through the hydrolysis of alkoxysilane precursors such as tetramethoxysilane and methyltrimethoxysilane, followed by condensation to yield a silica support [83–86].

Organic polymer-based monoliths have been employed in chiral separations [54,56,59,81,87–91]. A number of previous reports have prepared organic monoliths based on the use of glycidyl methacrylate (GMA) and ethylene dimethyacrylate (EDMA) [54,56,59,87]. These monoliths are relatively easy to prepare and can have high permeability and low back pressures, along with good efficiencies [81,92]. It is also possible to use these monoliths with various immobilization schemes to create protein-based CSPs [81,92].

Both AGP- and HSA-based CSPs have been prepared by using supports such as silica particles, silica monoliths, and GMA/EDMA monoliths and compared through the methods of frontal analysis and zonal elution [54,59,93,94]. Some typical results are shown in Table 2 [54]. In general, higher protein content has been obtained for these CSPs when using a silica monolith versus silica particles or GMA/EDMA monoliths. This higher protein content tends to result in higher retention and greater or comparable resolution for some chiral solutes when using AGP or HSA in the silica monoliths [54,59].

Zirconia is another material that has been used in protein-based CSPs [18,95,96]. Advantages of using porous zirconia as a chromatographic support include its good mechanical, chemical, and thermal stabilities [18,95,96]. This material is also stable over a pH range of 1–14 [18,95]. Zirconia has been considered for use in a BSA-based CSP [96]. A zirconia-containing mixed oxide (i.e., magnesia-zirconia) has also been used in BSA-based CSPs, with the latter type of support being reported to have a larger surface area, and better pore size distribution and pore structure than bare zirconia [95].

### 3.2. Development and Evaluation of Protein Immobilization Methods

Gentle methods are usually desirable for immobilization when making a protein-based CSP to maintain the conformation of the protein and its ability for chiral discrimination [18]. A number of immobilized proteins have been employed in CSPs, as is shown in Table 1 [18,23–26]. More details on each of these proteins are provided in Section 4. Commercial CSPs are available that make use of the proteins BSA, HSA, and AGP, as well as enzymes such as cellobiohydrolase I (CBH I) [17,18,20,97]. Immobilization methods for preparing these and other protein-based CSPs have included physical adsorption, covalent immobilization, and encapsulation [3]. The evaluation of methods for preparing protein-based CSPs has often been performed by using chromatographic techniques such as frontal analysis and zonal elution, as described in Section 2 [33–35,58,59].

#### 3.2.1. Physical Adsorption

Physical adsorption typically involves the simple adsorption of a binding agent to a surface [98]. Physical adsorption based on ionic and/or hydrophobic interactions has been employed to immobilize proteins such as lysozyme and avidin onto silica particles and monoliths to form CSPs, as well as in fused-silica capillaries [98–103]. Adsorption onto silica has also been used to make stationary phases containing BSA or its fragments [104], α-chymotrypsin [105], lysozyme [98], and avidin [99,100,106]. In addition, BSA has been adsorbed onto zirconia and used as a CSP [96]. In this last example, adsorption at pH 4.4 gave a greater amount of adsorbed protein and stronger retention than when pH 6.9 was utilized; however, a support made at pH 6.9 gave better stereoselectivity [96]. Anion-exchange silica materials and polyethylene hollow fiber membranes are other supports that have been utilized to adsorb BSA and HSA for use in CSPs [101,102].

The major advantage of physical adsorption is its simplicity. This method is generally carried out by simply pumping a protein solution through a column under appropriate pH and solution conditions for adsorption [18]. Such a technique can be employed for the rapid assessment of new protein candidates for use in CSPs [18]. One drawback of this immobilization method is that CSPs that are made by physical adsorption can have stability problems due to the presence of weak interactions between the adsorbed protein and support [18]. In some cases, the optimum conditions for a stereoselective separation may lead to the loss of an adsorbed protein over time, possibly requiring the use of crosslinking to prevent this loss [17].

#### 3.2.2. Covalent Immobilization

**Figure 4:**
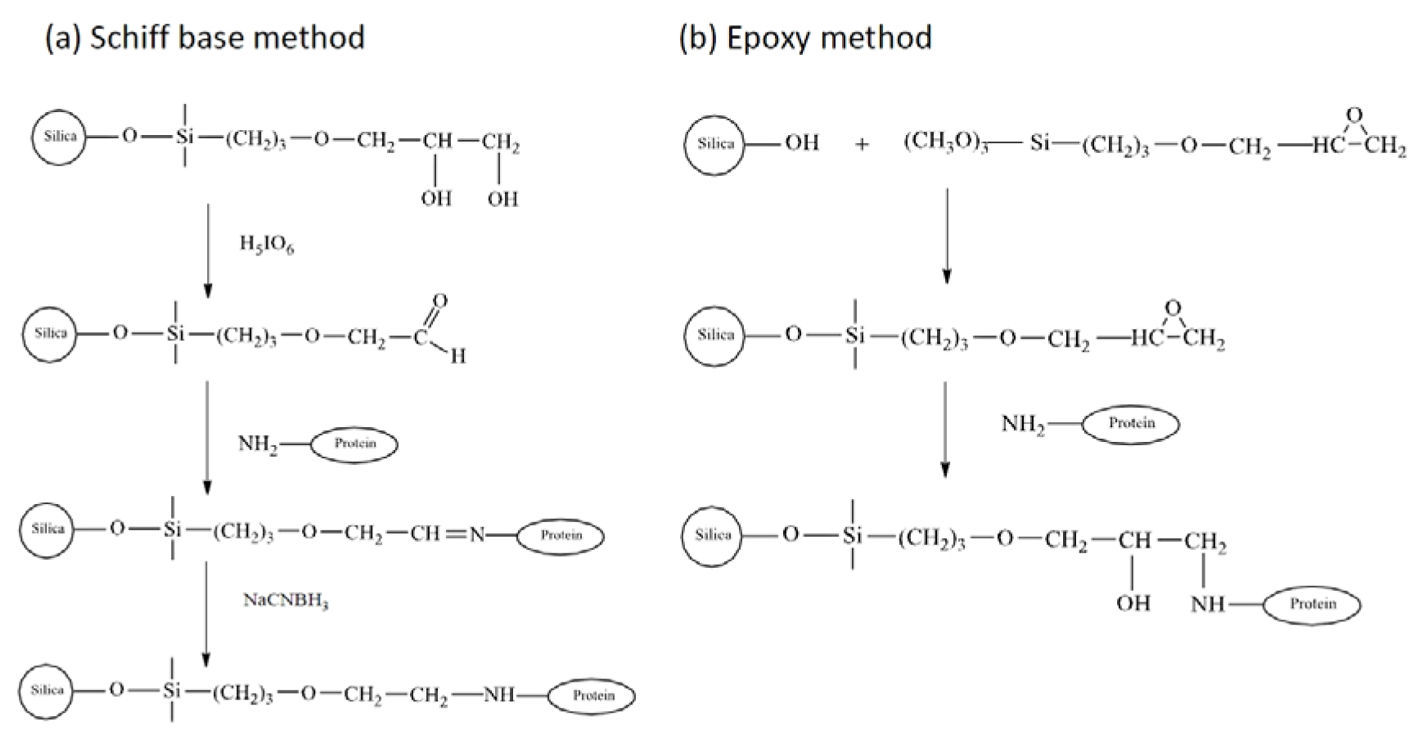
Examples of covalent immobilization methods for preparing protein-based CSPs on silica: (**a**) the Schiff base method and (**b**) the epoxy method.

Covalent immobilization is the most common approach for preparing protein-based CSPs and can result in supports with good long-term stabilities [18,107]. Many techniques have been used to couple proteins to supports in CSPs. Some of these techniques are the Schiff base method (i.e., reductive amination), the *N*-hydroxysuccinimide (NHS) method, the carbonyldiimidazole (CDI) method, the epoxy method, the ethyl dimethylaminopropyl carbodiimide (EDC) method, and the tresyl chloride method, as well as others [17,18,105,107]. Figure 4 shows a few examples of these methods.

A number of protein-based CSPs have been prepared using the Schiff base method. This is often done by taking a diol-containing support, oxidizing this support to form aldehyde groups, and then allowing these aldehyde groups to combine with free amine groups on a protein and in the presence of sodium cyanoborohydride as a mild reducing agent [18,55,71,108–110]. The Schiff base method has often been employed for preparing columns containing HSA or BSA as CSPs [22,45,53,57,59,65,72,104,111]. A modified form of the Schiff base method has been used to immobilize serum albumins to amine-containing silica that has been activated with glutaraldehyde [95,112]. Similar methods have also been used to immobilize antibodies onto silica for use in chiral separations [113,114].

The NHS method involves the use of a support that has been activated with a homo- or heterobifunctional agent such as *N*,*N′*-disuccinimidyl carbonate (DSC), *N*,*N′*-disuccinimidyl suberate (DSS), succinimidyl 4-(*N*-maleimidomethyl) cyclohexane-carboxylate (SMCC), or succinimidyl iodoacetate (SIC) [107]. DSC has been used to activate amine-derivatized silica for preparing various protein-based CSPs [3,17,33,35,109,115–118]. One report evaluated the binding of carbamazepine with HSA that had been immobilized by the Schiff base method or a DSC-based NHS method; these two approaches gave HSA columns with comparable binding constants and activities, but the NHS method gave less non-specific binding for carbamazepine [33]. However, CSPs that were prepared with amyloglucosidase and using DSC-activated silica showed poor stability when compared to a support made by the Schiff base method [110]. DSS has been used as an activating agent to immobilize HSA and avidin to aminopropyl silica for use as CSPs [109,115,116]. In one study, the retention and enantioselectivity of an avidin-based CSP made with DSS were found to be higher than the results that were obtained when using DSC [116].

The epoxy, CDI, and tresyl chloride methods are other approaches for immobilizing proteins through their amine groups [18,105,119–121]. The immobilization of α-chymotrypsin to silica has been examined when using the epoxy, tresyl chloride, and Schiff base methods [105]. The α-chymotrypsin-based CSP that was prepared by the epoxy method gave the highest enzyme content and the largest stereoselectivities in this group; a similar loading of the enzyme was observed in the other two methods, with the tresyl chloride method giving a support with lower stereoselectivity than that obtained with the Schiff base method [105]. Both BSA and HSA have been immobilized to diol-bonded silica particles by the CDI method, giving a final protein content that was two-fold higher than that obtained with the epoxy method [120]. The tresyl chloride method has also been used to immobilize BSA to silica, giving a comparable protein content to that obtained with the CDI method [120]. However, it has been noted for some drugs and solutes that the CDI method can result in greater non-specific binding than the Schiff base or NHS methods [33].

CSPs can be prepared by using other functional groups on proteins for immobilization. For instance, a protein can be coupled to a support through carboxylate groups by using the EDC method [17,122]. This method has been used to immobilize CBH I to amine-containing silica [122]. The same method has been employed for the immobilization of penicillin G acylase (PGA) [123]. Immobilization can also be achieved through the use of sulfhydryl groups on a protein. For instance, HSA has been immobilized through its lone free sulfhydryl group to amine-derivatized silica, which was activated by using SMCC or SIC [35]. HSA-based CSPs prepared by these methods were found to have comparable or improved activity and stability to HSA supports that were made by the Schiff base method [35]. In addition, it is possible to use carbohydrate groups to immobilized glycoproteins for use as CSPs [34,54]. An example is the use of mild oxidation to generate aldehyde groups in the carbohydrate chains of AGP, followed by the coupling of these groups with hydrazide-activated supports such as silica particles, silica monoliths, or GMA/EDMA monoliths [34,54].

#### 3.2.3. Encapsulation and Entrapment

When covalent immobilization is used, effects such as multisite attachment or the improper orientation of a protein can result in an actual or apparent loss of activity for this binding agent [107]. These effects can be avoided by instead using encapsulation or entrapment. These are non-covalent immobilization techniques based on the physical containment of a protein within a support [83–85,107]. Several reports have employed sol-gel encapsulation to prepare monoliths that contained BSA or ovomucoid for use as CSPs in capillary electrochromatography (CEC) [83,84,86]. This method has also been used to prepare a sol-gel/organic hybrid composite material containing encapsulated BSA [85].

An alternative method based on entrapment was recently reported in which HSA and AGP were entrapped in hydrazide-activated silica by using mildly oxidized glycogen as a capping agent [124–126]. This method has been used with both normal HSA and glycated HSA and has been found to give essentially full activity for these proteins in their binding with chiral solutes such as *R*-warfarin and L-tryptophan [125].

## 4. Chromatographic Studies of Protein-Based CSPs

### 4.1. Serum Transport Proteins

A number of serum proteins play a crucial role in transporting endogenous and exogenous compounds in the bloodstream, such as drugs, hormones, fatty acids, and metal ions [3,111,127]. Many of these interactions are also stereoselective in nature [3,128]. This feature has made serum transport proteins such as HSA, BSA, and AGP of interest for use as CSPs and in chromatographic studies of drug–protein interactions [3,128].

#### 4.1.1. Human Serum Albumin and Bovine Serum Albumin

HSA is the most abundant protein in human plasma (concentration, 30–50 mg/mL). This protein consists of 585 amino acids and has a molecular mass of 66.5 kDa [3,127]. BSA is the type of serum albumin found in cows [129] and is closely related to HSA, with a molecular mass of 66.2 kDa [3]. Both HSA and BSA are globular proteins with single polypeptide chains that are stabilized by 17 disulfide bridges [3].

HSA and BSA have two major drug binding sites that are often referred to as Sudlow sites I and II [130,131]. Sudlow site I (i.e., the warfarin-azapropazone site) is formed by a hydrophobic pocket in subdomain IIA of HSA [130,131]. Warfarin is commonly used as a probe for this site [132]. Sudlow site II (i.e., the indole-benzodiazepine site) is located in subdomain IIIA. Diazepam, L-tryptophan, and ibuprofen have all been used as probes for this region [133]. HSA and BSA also contain several binding regions for other solutes, such as fatty acids, metal ions, and other types of drugs or organic compounds [3].

The various sites on these proteins and the relatively broad range of solutes that can bind to these regions have made BSA and HSA of interest in obtaining chiral separations [3,17]. The use of serum albumins as binding agents for chiral separations has been widely examined in terms of the immobilization methods, types of supports and chromatographic conditions that can be used in such work [3,17,20]. Examples of chiral compounds that have been separated and studied by HSA- and BSA-based CSPs have included coumarins (e.g., *R/S*-warfarin) [119], benzodiazepine derivatives [43,134], barbiturates [135], benzothiadiazeines [135], α-arylpropionic acids (e.g., *R/S*-ibuprofen) [44], and amino acids (e.g., D/L-tryptophan) or hormones (e.g., D/L-thyroxine) and related derivatives [104,136–138].

Chromatography has been used to carry out thermodynamic and kinetic studies on the binding of chiral solutes such as *R*- and *S*-warfarin and D- and L-tryptophan with immobilized HSA columns (see Figures 2 and 3) [44,45,53,57,65,72]. For *R*- and *S*-warfarin, frontal analysis and zonal elution competition experiments indicated that these two enantiomers were binding to the same region on HSA but with a slightly higher binding capacity being observed for *R*-warfarin and a larger change in enthalpy being seen for *S*-warfarin [65]. Plate height measurements have been used to examine the interaction kinetics of *R*- and *S*-warfarin with immobilized HSA [72]. The dissociation rate constants for these interactions have been found to increase as the temperature is raised from 4 to 45 °C, with the value for *R*-warfarin going from 0.06 to 1.9 s^31^ and the value for *S*-warfarin increasing from 0.06 to 0.36 s^31^ [72]. The combined results obtained from these studies have further indicated that the chiral separation of *R*- and *S*-warfarin on an HSA column can be altered by varying the temperature [65,72].

Similar experiments employing frontal analysis and zonal elution have been used to investigate the binding of D- and L-tryptophan with immobilized HSA [45,53,57]. It has been found that these enantiomers bind to single but distinct sites on HSA, with L-tryptophan binding to Sudlow site II and D-tryptophan indirectly interacting with Sudlow site I [45,53,57]. The association equilibrium constants for D- and L-tryptophan with HSA were measured by chromatographic methods at temperatures ranging from 4 to 45 °C, with zonal elution studies also being used to estimate the dissociation and association rate constants for these interactions as a function of temperature, pH, ionic strength and solvent polarity [53,57]. In addition, studies with chemically-modified HSA have shown that HSA’s lone tryptophan residue (Trp-214) affects the stereoselective binding of HSA for solutes such as warfarin [64].

It has been found in chromatographic studies that the pH of the mobile phase can affect the retention and stereoselectivity of serum albumin-based CSPs. For acidic compounds like warfarin and carboxylic acids, a decrease in pH of a serum albumin-based CSP can lead to an increase in retention and a decrease in stereoselectivity [135,139]. For basic compounds that contain an amine group and are aromatic amino acids (e.g., D- and L-tryptophan), an increase in both the retention and stereoselectivity is observed on a serum albumin column when there is an increase in pH [3]. Ionic strength can also influence the retention of chiral compounds on these columns but does so in a complex manner because both electrostatic and hydrophobic interactions are affected when the ionic strength is altered [3,139]. Furthermore, the presence of an organic modifier such as 1-propanol can be used to adjust the retention and separation of chiral solutes on such columns [3,55,56].

In addition to supports like silica particles and agarose [3,17,140], monoliths have been used in serum albumin-based chiral separations [56,59,141,142]. For instance, an HSA-based CSP was made using a silica monolith and evaluated for its ability to bind and separate the enantiomers of warfarin, tryptophan, and ibuprofen; the results were then compared with those obtained on columns that contained HSA on silica particles or within GMA/EDMA monoliths [59]. Silica monoliths have also been modified with BSA and gold nanoparticles. This type of CSP was employed in capillary CEC to separate phenylthiocarbamyl amino acids [141]. Two versions of the Schiff base method were used to immobilize BSA onto silica monoliths for use in CEC, with these supports being compared for their ability to separate D- and L-tryptophan [142]. A GMA/EDMA monolith that contained HSA in affinity microcolumns was optimized and employed in chiral separations, making it possible to separate the enantiomers of warfarin and tryptophan within only 1.5–6.0 min [56].

**Figure 5:**
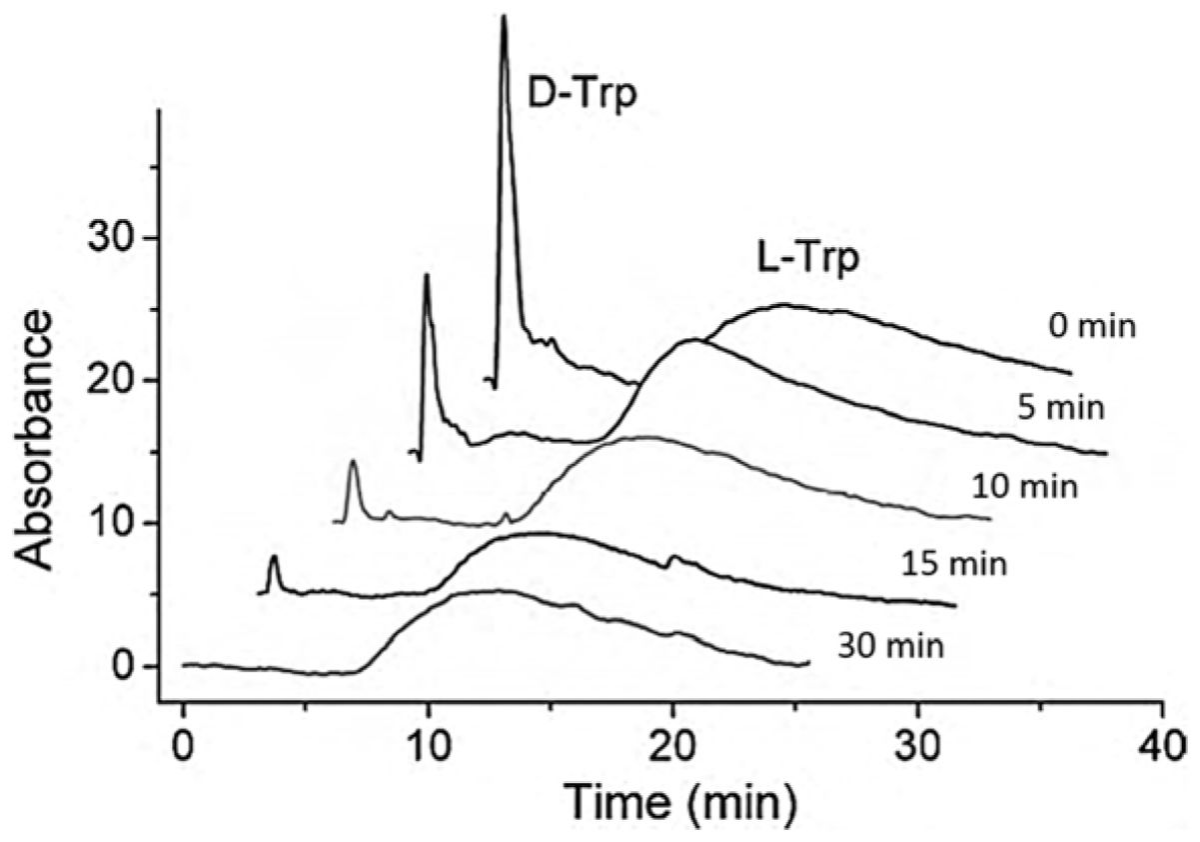
Chiral separation of D- and L-tryptophan and analysis of the oxidation of D-tryptophan by the enzyme D-amino acid oxidase using a column containing immobilized HSA. Reproduced with permission from [87]. Copyright Elsevier, 2010.

A number of other supports and column formats have been used to make serum albumin-based CSPs. Silica that contained both macropores and mesopores was used to immobilize BSA and tested for its ability to separate D- and L-tryptophan [143]. A chip-based column was made by placing dopamine and graphene oxide on the inner wall of an open-tubular capillary, with BSA then being immobilized to this coating. This column was used in CEC for the separation of D/L-tryptophan, D/L-threonine and a chiral dipeptide [144]. An HSA-based CSP has been used with affinity microcolumns and ultrafast affinity extraction to simultaneously measure the free, or non-protein bound, fractions of *R*- and *S*-warfarin in serum and to study the interactions of these enantiomers with soluble HSA [55]. Another study used an HSA-based CSP made on a GMA/EDMA monolith to study the oxidation of D-tryptophan by the enzyme D-amino acid oxidase (see Figure 5) [87].

#### 4.1.2. Alpha_1_-Acid Glycoprotein

AGP is a glycoprotein that has a normal concentration in human serum of 0.5–1 mg/mL [145,146]. Human AGP has an average molecular mass of 41 kDa and consists of a single chain of 183 amino acid. This protein has an isoelectric point of 2.8–3.8 [145,146] and contains five *N*-linked glycan chains, giving it a carbohydrate content of 45% (*w*/*w*) [145,147,148]. AGP is an important transport protein in the blood for many basic and neutral drugs, as well as some acidic drugs (e.g., warfarin) [145,147,149–151]. AGP has a highly symmetrical β-sheet structure that is comprised of an eight-stranded β-barrel [146,147,152]. This protein is believed to have one wide and flexible drug-binding region that is common for basic, acidic, and neutral drugs [150,152,153].

Thermodynamic studies have been conducted by chromatography and with immobilized AGP to examine the interactions of *R*- and *S*-propranolol with this protein [34,58,154]. The binding of both enantiomers to AGP was found to be mainly an enthalpy-driven process [34]. In addition, frontal analysis experiments have suggested that the interaction of *R*- and *S*-propranolol with AGP followed a saturable/nonsaturable mixed-mode model, with the saturable site having an equilibrium constant of (1.1 ± 0.6) × 10^6^ M^31^ for *R*-propranolol and (1.4 ± 0.7) × 10^6^ M^31^ for *S*-propranolol at 37 °C [154].

Several reports have employed commercial AGP-based CSPs to evaluate the effects of changing the mobile phase composition and column temperature on the resolution or retention of various chiral solutes [155,156]. Altering the mobile phase pH and column temperature can be used to control the elution order of some chiral solutes and to resolve multiple chiral compounds by using gradient elution [155]. The use of organic modifiers such as methanol, ethanol, propanol, and tertiary alcohol additives have also been investigated in terms of their effects on the stereoselectivity of AGP columns [156].

Commercial AGP columns have been used for the separation of many chiral compounds [154,157–164]. For example, chiral separations have been achieved by using these columns for atenolol, citalopram, warfarin, verapamil, norverapamil, atropine, and vinca alkaloid analogues [157–161]. Liquid chromatography-tandem mass spectrometry (LC-MS/MS) has been used with AGP columns for the stereoselective separation and quantitation of drugs such as methadone [162] and ornidazole [163].

Work has also continued in the development of new methods and supports for preparing AGP-based CSPs [54,58]. One type of AGP-based CSP was prepared by mildly oxidizing the carbohydrate chains on AGP and using these modified residues to immobilize this glycoprotein onto hydrazide-activated silica [34,58]. A similar approach has been used to prepare CSPs in which AGP was immobilized to monoliths based on silica or GMA/EDMA [54].

### 4.2. Enzymes

#### 4.2.1. Penicillin G Acylase

PGA is an *N*-terminal nucleophile hydrolase that is widely present in bacteria, yeast, and fungi [23,165]. Mature PGA from *E. coli* is a heterodimer of 20.5 kDa and 69 kDa subunits that contain 209 and 557 amino acids, respectively [23]. PGA is often used in the pharmaceutical industry for hydrolytic cleavage of the acyl chain of penicillin G [23,165,166]. However, this enzyme has also been found to be useful as a CSP for a variety of acidic aromatic compounds because of its binding specificity for acyl groups [165,166]. Other attractive features for PGA as a chiral binding agent are its low cost and availability in a highly pure form [123].

PGA has been studied in several reports as a possible CSP. For instance, the chiral recognition mechanism of PGA has been examined, by chromatography and other methods, for a series of 2-aryloxyacetic acids [166,167]. PGA has been immobilized to silica through amine or carboxylate groups and employed in an HPLC system for both hydrolyzing penicillin G and carrying out a chiral separation on the products [123]. Another report used a PGA-based silica monolith in capillary liquid chromatography for the chiral resolution for arylpropionic acids such as ketoprofen [168].

#### 4.2.2. Cellobiohydrolases

Cellobiohydrolases (CBHs) are enzymes found in fungal cellulose systems that have the ability to hydrolyze β-1,4-glycosidic bonds [17,169]. The fungus *Trichoderma reesei* can produce two major types of CBHs (i.e., CBH I and II), both of which are naturally *N*- and *O*-glycosylated [169]. Of these two types, CBH I has been employed the most as a chiral selector. This enzyme has been used in chiral separations to resolve acidic, basic, and neutral compounds [3,17,170]. CBH I consists of nearly 500 amino acids that make up a large core, a small cellulose-binding domain, and a flexible connecting arm [17,170–172]. The catalytic domain of the core is the area that provides the chiral selectivity for this enzyme [3,173,174].

The largest stereoselectivity for CBH I has been observed in the separation of β-blockers like propranolol, alprenolol, oxprenolol, and metoprolol [3,17]. This observation agrees with the fact that propranolol and alprenolol can inhibit CBH I stereoselectively, with the *S*-enantiomers having stronger inhibitory effects and affinity than the *R*-enantiomers [174]. A number of reports have used commercial CBH-based CSPs with LC and MS detection [169,175,176]. For example, a CBH-based CSP has been used with LC-MS/MS to analyze 56 drug biomarkers in wastewater, including 18 pairs of enantiomers for drugs such as amphetamine, methamphetamine, 3,4-methylenedioxy-methamphetamine, and their metabolites [176].

#### 4.2.3. α-Chymotrypsin

α-Chymotrypsin (sometimes referred to as “α-CHT” or “ACHT”) is a digestive enzyme and serine protease [3]. This enzyme can hydrolyze a peptide chain on the *C*-terminal side of residues that contain aromatic amino acids (e.g., tyrosine, phenylalanine or tryptophan) [3]. This enzyme has three polypeptide chains and consists of a total of 241 amino acids [177]. The substrate-binding site of this enzyme is composed of a hydrophobic pocket and a hydrolytically active site that is formed by Ser 195, His 57, and Asp 102 [178].

This enzyme has been covalently immobilized to silica and used as a CSP for the stereoselective resolution of various amino acids, amino acid derivatives, and dipeptides [179]. Aromatic amino acids such as tyrosine, phenylalanine, and tryptophan, which are recognized by the substrate-binding site of α-chymotrypsin, have been the main targets of interest in these studies [3,17]. It has been found that the *O*- and *N*-derivatives of these amino acids (e.g., methyl esters or amides) can also be resolved by using this type of CSP [105]. In addition, α-chymotrypsin has been used in chiral separations for some amino acids that are not substrates for this enzyme (e.g., leucine and alanine) [3]. α-Chymotrypsin-based CSPs have further been used to separate a series of enantiomeric and diastereomeric dipeptides [180]. In addition, amino acid derivatives and chiral compounds such as naproxen and aryloxyproionic acids have been separated by using α-chymotrypsin-based CSPs [121].

### 4.3. Other Proteins

A number of other proteins have been considered as binding agents and CSPs for chromatography. One example is ovomucoid, which is a glycoprotein found in egg whites and that contains three homologous domains [118]. Ovomucoid columns have been used for chiral separations of a large number of drugs and chiral solutes [28,118,164,181–184]. In one recent study, an ovomucoid-based CSP was used with LC-MS/MS to separate and analyze the enantiomers of ondansetron and doxazosin in human plasma and to give information on the pharmacokinetics of these agents [185,186].

**Figure 6:**
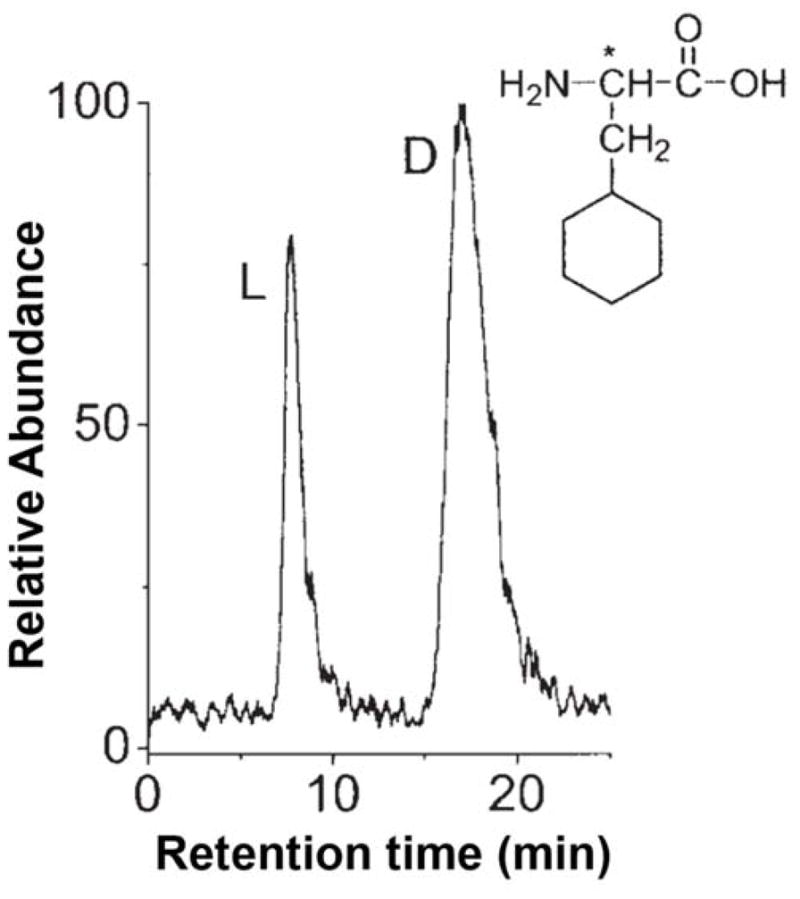
Chiral separation of D- and L-3-cyclohexylalanine on a column containing immobilized anti-D-amino acid antibodies. Adapted with permission from [192]. Copyright John Wiley and Sons, 2006.

Antibodies, which are glycoproteins that are produced by the immune system of vertebrates, are another group of proteins that have been explored for use in chiral separations [187–193]. Various anti-α-amino acid antibodies have been used as chiral agents to separate aromatic and aliphatic amino acids (see Figure 6) [189,191,192]. Antibodies against α-hydroxy acids have been employed to resolve the enantiomers of several α-hydroxy acids, including both aromatic and aliphatic compounds (e.g., lactic acid) [193]. In one report, a monoclonal anti-D-hydroxy acid antibody was used to isolate and analyze the enantiomers of lactic acid in human urine by using immunoaffinity chromatography and LC-MS [190].

Avidin is a glycoprotein that is obtained from egg whites and that can strongly bind to biotin [194]. Avidin-based CSPs have been investigated for use with drugs and solutes that have included thalidomide, glutethimide, premaquine, aminoglutethimide, and pyridoglutethimide [195]. In addition, this protein has been used in open tubular columns and in CEC for chiral separations [99,100,106,196–198]. Avidin-based CSPs have also been prepared by immobilizing this protein to a phospholipid coating or to magnetic particles in a fused silica capillary [106,197]. Chiral separations have been obtained with avidin in open tubular columns for a variety of chiral compounds, including ketoprofen, flurbiprofen, ibuprofen, abscisic acid, chrysanthemic acid, warfarin, tryptophan, omeprazole, and 5-hydroxyomeprazole [99,100,106,196–198].

Streptavidin is a biotin-binding protein that is produced by the bacterium *Streptomyces avidinii* [20,26]. Streptavidin and avidin are both tetrameric proteins and have similar primary structures, but the overall charges for these two proteins are quite different, with streptavidin having an isoelectric point of 5 and avidin having a value of 10 [20,194]. Streptavidin has been employed as a CSP on a perfusion support for separating adenosine enantiomers [199]. The role of the biotin binding sites in this process was examined by chromatography, as well as the binding capacity for D- and L-adenosine and the effects of sample concentration and temperature on solute retention [199].

## 5. Conclusions

The separation and analysis of chiral drugs has become important in both the discovery and development of new pharmaceutical agents [7,9]. Chromatographic methods employing proteins as chiral selectors have been found in recent years to be powerful tools for separating chiral drugs or solutes and for examining their interactions with proteins [6,9,17–19]. This review gave an overview of the chromatographic studies and applications that have involved protein-based chiral separations. Several chromatographic approaches were discussed that have been used to characterize and examine these separations, including zonal elution, frontal analysis, and kinetic methods of analysis. Supports and immobilization methods that have been used to prepare protein-based CSPs were also summarized. Finally, examples of proteins that have been considered for use in CSPs for chromatography, and the applications of these proteins were considered. These proteins ranged from serum transport proteins and enzymes to other proteins such as ovomucoid, antibodies, and avidin or streptavidin [17,18,20]. These protein-based CSPs have already been employed with a wide range of chiral drugs and solutes, as was illustrated by the examples given within this review. It was also shown how chromatographic methods have been used to provide valuable information for better characterizing and optimizing these chiral separations [17,18,20]. Future work in this area and in the use of chromatography with protein-based CSPs should lead to the continued development of improved chiral separations for drug discovery or development and pharmaceutical research [9,82,97].

## Figures and Tables

**Table 1:** Examples of proteins that have been used as chiral stationary phases for liquid chromatography a. pI: isoelectric point; CBH: cellobiohydrolase.

Type of Protein	Molecular Mass (kDa)	Isoelectric Point, pI
*Serum transport protein*		

α_1_-Acid glycoprotein (AGP)	41	2.7
Human serum albumin (HSA)	66	4.7
Bovine serum albumin (BSA)	66.5	4.7–4.9

*Enzymes*		

Cellobiohydrolases		
CBH I	60–70	3.9
CBH II		
α-Chymotrypsin	25	8.1–8.3
Lysozyme	14	11
Penicillin G acylase	90	6.4

*Other proteins*		

Antibodies (e.g., immunoglobulin G)	~150	4.4–10
Avidin	68	10
Ovomucoid	28.8	3.9–4.5
Streptavidin	60	5

a This table is based on information that was obtained from Refs. [18,23,24].

**Table 2:** Properties of AGP immobilized to various supports and chromatographic parameters of these supports for *R*- and *S*-propranolol^a^.

Type of Support	Silica Monolith	Silica Particles	GMA/EDMA Monolith
Protein content (nmol/g support)	1800 (± 40)	490 (± 20)	250 (± 10)
Protein coverage (nmol/m^2^)	5.8 (± 0.2)	4.9 (± 0.2)	3.6 (± 0.1)
Retention factor, *k*	31.1 (± 0.4) (*R*)	11.6 (± 0.2) (*R*)	4.53 (± 0.06) (*R*)
	37.2 (± 0.4) (*S*)	13.7 (± 0.3) (*S*)	6.86 (± 0.05) (*S*)
Separation factor, *α*	1.20 (± 0.01)	1.18 (± 0.05)	1.51 (± 0.06)
Resolution, *R_s_*	1.11 (± 0.03)	0.55 (± 0.03)	0.79 (± 0.03)

a Adapted with permission from [54]. Copyright Elsevier, 2007.
